# Stability of Alkaloids during Drying Process and Their Effect on Anticoagulating Activity of *Uncariae Ramulus Cum Uncis*

**DOI:** 10.1155/2019/7895152

**Published:** 2019-01-03

**Authors:** Lu Zou, Fengyu Lu, Bing Lin, Ying Zhou, Tingting Liu, Yue Sun

**Affiliations:** ^1^School of Pharmacy, Guizhou University, Guiyang, Guizhou, China; ^2^Guiyang College of Traditional Chinese Medicine, Guiyang, Guizhou, China

## Abstract

The drying process of *Uncariae Ramulus Cum Uncis* (URCU), a kind of traditional Chinese medicine, was studied in a scale dryer in laboratory at 65°C. It was observed that the alkaloids content of URCU firstly showed a tendency of increasing and then decreasing after reaching the peak at the 570th minute in the process of constant temperature drying. Moreover, the coagulation time of rabbit determined by test tubes has been adopted to study the effect imposed by the content of alkaloids on the anticoagulating activity of URCU. In addition, the software of Minitab was also utilized to fit the correlation between the content of alkaloids and the anticoagulating activity of URCU. The results obtained demonstrated that anticoagulant activities were available in both *rhynchophylline* and *isorhynchophylline*, among which the latter was the stronger one, while procoagulant activity was shown in *corynoxeine*. The case study can provide a useful reference for the research on drying other Chinese herbal medicines (CHMs) and further study on URCU.

## 1. Introduction

It is known worldwide and acknowledged that Chinese herbal medicines (CHMs) have been employed in the therapy of many diseases for several thousands of years in both China and neighbor countries and they play an increasingly important role in modern pharmaceutical industry [1]. Moreover, due to their great bioactivity and low toxicity, they have attracted much attention from western countries [2]. In addition, Liang et al. stated that CHM has great potential in overcoming some incurable diseases, for instance, cancer [3]. Therefore, many research groups have started their projects on CHM and have made some great contributions.

As an important analytical method, high-performance liquid chromatography (HPLC) has been greatly improved in several fields over the years. For instance, HPLC plays an important role in determining contents, analyzing constituents of many compounds, and obtaining the HPLC spectrum of CHM, which are deemed as useful in the identification and quality control of botanical medicines [4–6]. However, it was found that multicomponents instead of one contribute to the active compounds of CHM [7]. Xu's group suggested that the HPLC spectrum can help analyze the whole fingerprint of CHM, which illustrates the detailed compounds of a CHM [8]. Additionally, the appearance of spectrum-effect relationship provides a method to identify the main active compounds of CHM, which is of vital importance in development of CHM.

**Figure 1:**
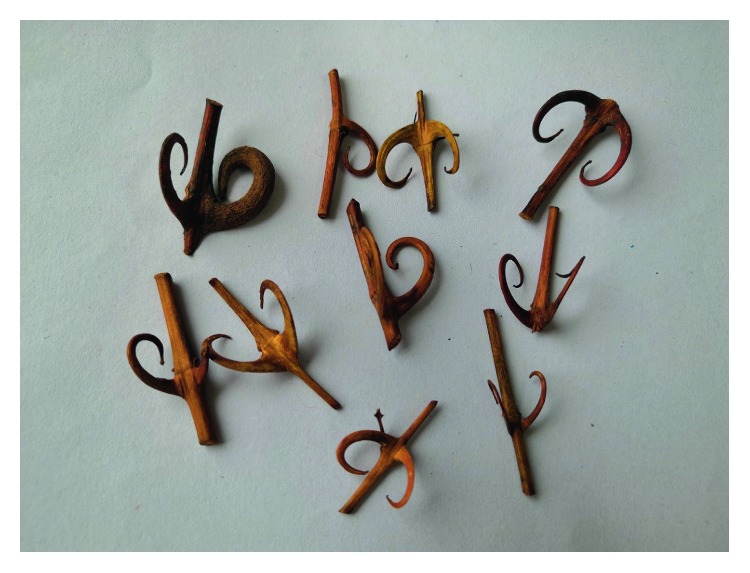
The dry stem with hook of URCU.

As shown in Figure 1, *Uncariae Ramulus Cum Uncis* (URCU) is the dry stem with hook of *Uncaria rhynchophylla* (Miq.) Miq. ex Havil., *Uncaria macrophylla* Wall., *Uncaria hirsuta* Havil., *Uncaria sinensis* (Oliv.) Havil., *or Uncaria sessilifructus* Roxb. [9]. Generally speaking, Jiangxi Province, Guangdong Province, Guizhou Province, and Guangxi Province of China are home to these plants [9]. Additionally, it is also deemed as a regional medicine of Guizhou Province in China. It is shown by pharmacological investigation that URCU has the function of clearing heat and soothing liver activity [10]. Apart from that, it can also be used to treat headache, vertigo, eclampsia during pregnancy, and cardiovascular disease to certain extent [11]. Through chemical studies, it was shown that the main active compounds are alkaloids, which contain mainly the active ingredients of *rhynchophylline*, *isorhynchophylline*, and *corynoxeine* [12]. They are quite beneficial to lowering blood pressure [13]. Besides, alkaloids of URCU are known for antiarrhythmic and antiepileptic aggregation, as well as antithrombotic effects [13–15]. Chen et al. proved that *rhynchophylline* can inhibit not only platelet aggregation agents such as thrombin but also the release of other active substances from the platelet membrane, indicating that its function is diversified [16].

Among the processing methods employed for CHM, drying which is known as the oldest way is one of the most important methods to remove the moisture from the solid. Moreover, this method is the key factor that would affect the quality of CHM. However, theoretical study on the drying process of CHM has been rarely reported so far. The quality criteria of most CHMs are empirical, and therefore, their quality in the aspects of their appearance, color, and smell are often criticized. However, less attention was paid to the variation of effective substances in CHM in the drying process. Our group has conducted studies on the most suitable drying model, as well as the kinetic parameters in the drying process for URCU [17]. But, the content variation of the alkaloids during the drying process and its bioactivity is still not clear. Hence, this paper demonstrates the variation of alkaloids content and the relationship between the alkaloids content and its anticoagulating activity during the drying process of URCU. In all, this case study can provide a useful reference for the study on drying process of CHM and further study on URCU.

## 2. Materials

### 2.1. Materials

URCU was collected from Guangxi Province of China in April 2016 and identified by Prof. Shenghua Wei (Guiyang College of Traditional Chinese Medicine). The standards of *isorhynchophylline* (151127), *rhynchophylline* (151018), and *corynoxeine* (151102) were purchased from the Xi'an Tianbao Biological Technology Company (Xi AN, China). HPLC‐grade methanol (Thermo Fisher) and analytical‐grade ammonia were purchased from Fuyu Refined Chemistry Company (Tianjing, China). As for the water used for HPLC analysis, it was purified by using a Milli‐Q water purification system (Millipore, MA, USA).

### 2.2. Experimental Instruments

To record the weight loss of samples, an analytical balance (model FA2004, Liangping, Shanghai, China), with a sensitivity of 0.0001 g, was employed. The drying apparatus used was an electric thermostatic dryer of vertical type (model DGG-9246A, Xinqi, Shanghai, China), which basically consists of a centrifugal fan for the supply of air flow, an electric heater, an air filter, and an electronic proportional controller. As for the proportional controller, it was used mainly to control the air temperature, while the centrifugal fan and fan speed control unit were used to regulate the air velocity. The air was firstly passed from the heating unit to be heated to the desired temperature, and then it would be channeled to the drying chamber. The samples were dried in the perforated square chamber, which had a flow cross section. Some other instruments include a grinder (model FW100, Tyster, Tianjing, China), a shaking table (model LZ-052, Zhanxin Instrument Company, Shanghai, China), and an Agilent 1220 series HPLC (Agilent Technologies, MA, USA) which was equipped with an online degassing system, a binary pump, an auto sampler, and a thermostatted column compartment which were all connected to the Agilent Chem Station. Moreover, there were also other instruments including a ZORBAX SB‐C_18_ column (150 × 4.6 mm, 5 *μ*m; Agilent Technologies, MA, USA), an ultrasound system (model SG8200HR, Guangte Ultrasound Instrument Company, Shanghai, China), and a rotary evaporator (model RE-52CS, Yarong, Shanghai, China). In addition, the other instruments required also included rabbit fixator, scissors, disposable lancet, anticoagulant tube, coagulation tube and timer, 0.45 *μ*m microporous membrane, syringe, and medical alcohol.

### 2.3. Experimental Animals

Animals used were rabbits, male, with a bodyweight of 2.5 ± 0.5 kg, provided by Chongqing Tengxin Biotechnology company (Chongqing, China); its bones are thick, with full muscles, limbs are normal gait, back hair is luster and rich, flexible behavior, the posture of head and neck is normal, eyes are bright, and no eye or nose discharge.

## 3. Methods

### 3.1. Measurement of Content Variation

#### 3.1.1. Drying Samples

The samples firstly were dried at air temperature of 65°C, and then, the accurately weighed (30.00 g) samples were distributed uniformly onto the plate (samples thickness of around 2.0 cm) of the dryer after the dryer reached steady-state conditions of the set points (at least 30 min) [1]. The drying time periods were 0, 30, 80, 140, 270, 400, 570, 800, 1440, 2160, 2880, 3600, and 4320 min, respectively. In addition, more than three samples at each certain time point were taken out, and then they were crushed into powder for storage.

#### 3.1.2. HPLC Conditions

Chromatographic separation was conducted on a ZORBAX SB‐C_18_ column (150 × 4.6 mm, 5 *μ*m), and the temperature was maintained at 30°C [7]. The detection wavelength was at 245 nm. With a sample injection volume of 10 *μ*L, the flow rate was 1.0 mL/min. The mobile phase was methanol-0.2% aqueous ammonia (70 : 30) with isocratic elution for 20 min.

#### 3.1.3. Preparation of Standard Solutions

After accurately weighing 5 mg *isorhynchophylline*, *rhynchophylline*, and *corynoxeine*, respectively, the three compounds were added to the three 10 mL volumetric flasks, respectively, and dissolved in the distilled water.

#### 3.1.4. Preparation of Sample Solutions

*(1) Preparation of Sample Solution in Different Dry Conditions*. Samples of 15 g, whose drying time periods are 0, 30, 80, 140, 270, 400, 570, 800, 1440, 2160, 2880, 3600, and 4320 min, respectively, were ultrasonically extracted for three times with 150 mL aqueous methanol with a concentration of 70% for 1 h. Then, filtrates from each extraction were consolidated and concentrated to the state where all methanol is gone under vacuum. After that, the residue liquid was kept and stored. Before the experiment, distilled water should be added into the residue liquid to make up to 30 mL, which made sure that the concentration of the sample was 0.5 g/mL.

#### 3.1.5. Determination of Blood Coagulation Time

After taking 1 mL blood from the rabbit's ear vein, gently put it into the test tube along the wall of test tube (the tube with a diameter of 8 mm and a length of 15 cm is required to clean) [18]. Furthermore, 0.1 mL extract solution of the dry sample, whose drying time periods are 0, 30, 80, 140, 270, 400, and 570 min, respectively, were added to 7 tubes, respectively [18]. Then, all of them were shaken by the shaking table for 1 min to mix the anticoagulant compounds well with the blood. When the blood solutions were sucked into the syringes, the recording process was conducted with the stopwatch. Generally, the test tubes were tilted at an angle of 30° at every 30 s to see if the blood flowed. When the blood no longer flowed at the time the test tube was tilted to 90°, it was completely solidified and this operation was stopped. And thus, the coagulation time refers to the time at the finishing moment. This experiment was conducted at 15°C. The longer the blood coagulation time is, the stronger the anticoagulating activity of the sample is.

### 3.2. Analysis

#### 3.2.1. Analysis of HPLC Spectrum

Analysis was conducted for both the standard solutions and sample solutions obtained in Sections 3.1.3 and Preparation of Sample Solution in Different Dry Conditions in Section 3.1.4, respectively at the 80th min to determine the precision [7]. Through three injections of one sample, the repeatability was evaluated and each sample solution was detected three times in parallel.

#### 3.2.2. Partial Least-Squares Regression Analysis

In the process of partial least-squares analysis, the Minitab statistical software was employed to calculate the distribution of residual [3]. Besides, Minitab Statistical Software was also used to deal with the relationship between the coagulation time of the sample and the peak areas, which are subjected to regression analysis by partial least squares.

## 4. Results and Discussion

### 4.1. The HPLC Spectrum of the Standard Solution and Decoction of Sample

**Figure 2:**
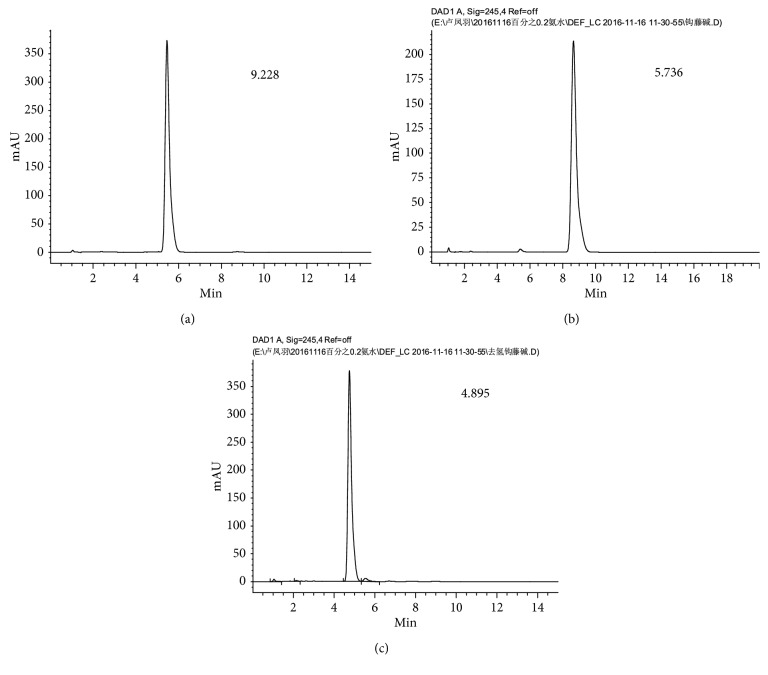
HPLC spectrum of standard solution. (a) *Rhynchophylline*, (b) *isorhynchophylline*, and (c) *corynoxeine*.

**Figure 3:**
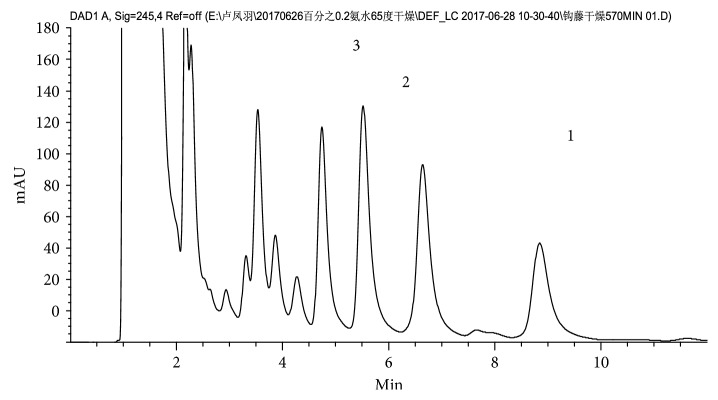
HPLC spectrum of drying process of sample collected at 80 min (1, *rhynchophylline*; 2, *isorhynchophylline*; 3, *corynoxeine*).

The retention time periods of the three alkaloids (*rhynchophylline*, *isorhynchophylline*, and *corynoxeine*) are 9.288, 5.376, and 4.485 min, respectively, as shown in Figure 2. The HPLC spectrum of the sample during the drying process collected at the 80th minute is presented in Figure 3.

### 4.2. The Measurement Results Obtained from the Sample Solution in Different Drying Conditions

**Figure 4:**
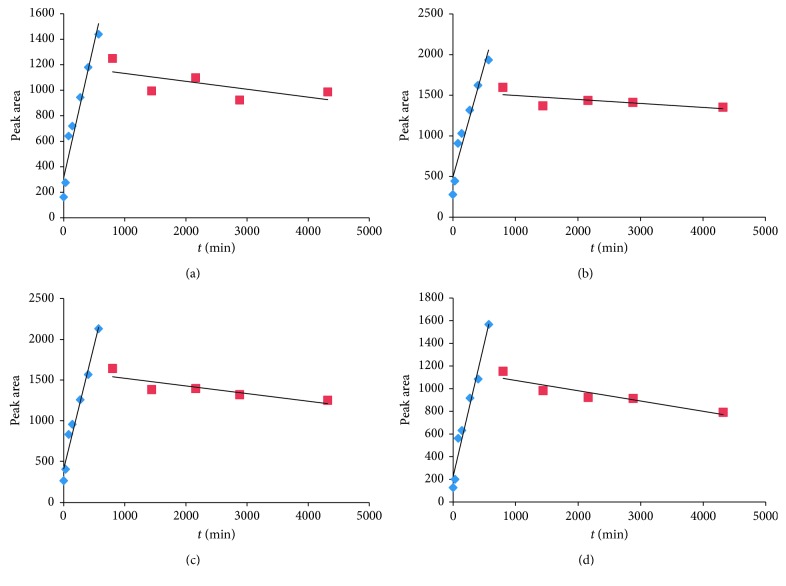
The variation of the content of each characteristic component with time. (a) *Rhynchophylline*, (b) *isorhynchophylline*, (c) *corynoxeine*, and (d) the sum of peak areas.

The peak areas shown in the HPLC spectrum of water extracts from 13 samples prepared in Section 3.1.4 were obtained. Moreover, the peak areas and their corresponding drying time periods were plotted according to the results obtained. As shown in Figure 4, the variation of the content of each alkaloid along with the passage of time in the drying process can be gained, which indicated that the content of the three alkaloids showed a trend of increasing firstly, achieving the peak at the 570th minute and then decreasing.

Statistical software was used to solve the linear fitting analysis of peak areas obtained from the three alkaloids and the sum of peak areas in the rising phase and the descending phase in Figure 4.  Table 1 shows the fitting function and the correlation coefficient obtained in the study. Good linear relationships between the peak areas of the three alkaloids and the drying time in the rising phase (all correlation coefficients *R* are >0.9) are shown in Figure 4 and Table 1. In the descending phase, although the correlation coefficient of the fitting was slightly low, it could still approximate to that of the first-order kinetic process. According to the fitting equation, the content gained from each of the alkaloids can be calculated approximately at any time with a drying temperature of 65°C.

### 4.3. Anticoagulant Experiment

**Figure 5:**
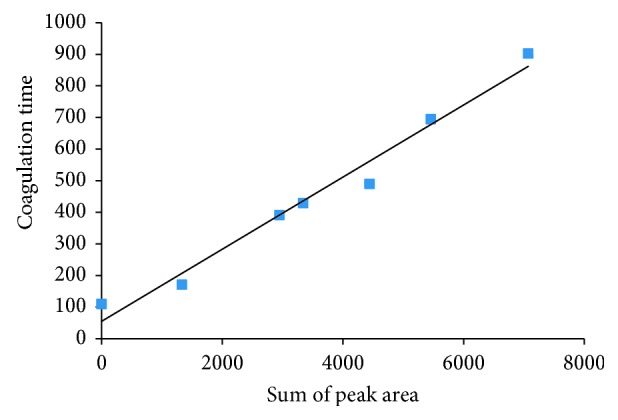
Relationship between peak area and coagulation time of dried samples.

Under the drying condition of 65°C, when the drying time reached about 200th minute, the moisture content of URCU satisfied the requirements of the Chinese pharmacopoeia [17]. Therefore, the samples in the initial stage during the drying process (the rising stage of alkaloids content) were selected to study the correlation between the variation of alkaloids and anticoagulant activity. In the rising stage of alkaloids content at the constant temperature of 65°C, the sum of peak area of three alkaloids of the sample indicated an increasing trend by prolonging the drying time, and the anticoagulant activity of the corresponding sample also showed a gradual increasing trend at the same time. As shown in Figure 5, the sum of peak area versus coagulation time tended to be a straight line.

It can be seen from Figure 5 that there was a positive correlation between the peak area and the coagulation time of the blood sample when the correlation coefficient *R* ^2^ equaled to 0.9888. The fitting equation is shown in equation (1) as follows:

(1) $$\begin{array}{c} Y=0.1708X+19.206, \end{array}$$

where *X* refers to the sum of peak area, while *Y* refers to the coagulation time.

### 4.4. Analysis Results Obtained from Partial Least-Squares Regression

**Figure 6:**
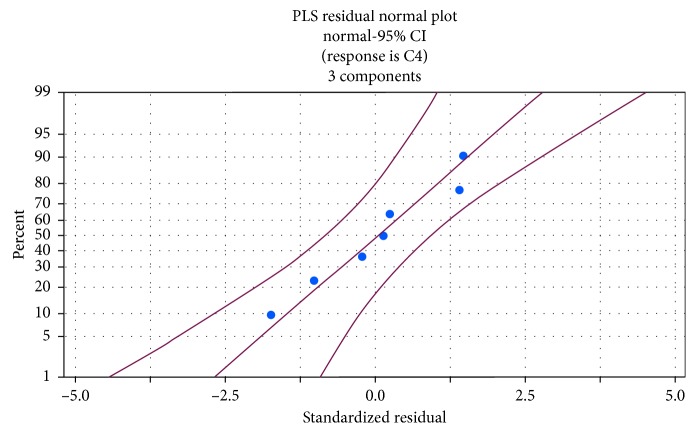
Model residual normal distribution graph.

The coagulation time in this study was regarded as the dependent variable. From the figure of state distribution (as shown in Figure 6), it can be shown that the standardized residuals of the model were basically distributed in the vicinity of the fitted line, which means that the residuals can generally satisfy the characteristics of normal distribution, and hence the fitted model can be considered as reasonable.

As for the analysis of the partial least-squares regression model, the degree of the model fitting is mainly shown in the form of the value obtained from the correlation coefficient (*R* ^2^). The closer the value of *R* ^2^ is to 1, the better the model fits. In this study, the correlation coefficient gained from the best model selected during the study was *R* ^2^ = 0.9976; therefore, the correlation between *X* and *Y* could be considered as quite good.

Furthermore, the correlation between the coagulation time and the peak area of the sample was obtained as well and the equation of the spectrum effect was established in equation (2) as follows:

(2) $$\begin{array}{c} Y=0.058X1+0.924X2-0.698X3-61.7, \end{array}$$

where *Y* is the coagulation time, *X*1, *X*2, and *X*3 are the peak areas of *rhynchophylline*, *isorhynchophylline*, *corynoxeine*, respectively.

It can be seen from equation (2) that the three alkaloids of the sample showed different performance on the anticoagulant activity. In detail, *rhynchophylline* and *isorhynchophylline* showed anticoagulant activity, among which the latter is stronger, while *corynoxeine* indicated procoagulant activity.

## 5. Conclusion

In the field of agricultural products, mathematical modeling of the drying process was widely used [18–21], but its application to traditional Chinese medicine was rare. Besides, although previous studies on the most suitable model of URCU and its related parameters are available, the variation existing in the content of three alkaloids in URCU and their bioactivity during the drying process is still not clear. Therefore, this paper focuses on the variation of content and its anticoagulating activity. It was shown by the study that the alkaloids content of URCU firstly showed a tendency of increasing and then decreasing after arriving the highest point at the 570th minute in the process of constant temperature drying. It was reported that three compounds disappeared and two new elements appeared from the HPLC spectrum of URCU decoction collected at different time points [22]. It demonstrated that the extracts of URCU could be transformed mutually in the continuous heating process, which might be the reason why the content of alkaloids increased in the initial stage. However, degradation resulted in a drop in the content of alkaloid with the prolongation of heating time.

During the drying process, the peak areas and the contents of the three alkaloids showed the same trend basically, but the three alkaloids indicated subtle differences. Therefore, the peak areas of several alkaloids in the HPLC spectrum could be considered as the basis for criticizing the quality of URCU during the drying process (no content testing is specified in Chinese pharmacopoeia). A good linear relationship was shown between the content of alkaloids and drying time at the rising phase of the drying process (correlation coefficient *R* is above 0.9). Through the analysis of the linear relationship, it is most likely to roughly estimate the content of the alkaloids at any time during the drying process.

Finally, in the drying process of URCU, there was a good positive correlation among the drying time of the sample, the sum of peak areas of alkaloids and the anticoagulant activity in vitro. Thereby, the sum of peaks of three alkaloids might be further considered as quality criteria of URCU in drying process. However, after employing Minitab, the result showed that the three alkaloids indicated different anticoagulant activities of the samples. In detail, *rhynchophylline* and *isorhynchophylline* had anticoagulant activity, among which the latter was stronger, while *corynoxeine* represented procoagulant activity.

## Figures and Tables

**Table 1:** The relationships between peak areas and drying time and its correlation coefficient (*R* ^2^) of three alkaloids during drying process.

Alkaloids	Fitting equation (rising phase)	*R* ^2^	Fitting equation (descending phase)	*R* ^2^
*Rhynchophylline*	*y*=2.12*x*+315.56	0.937	*y*=−0.10*x*+1264.90	0.810
*Isorhynchophylline*	*y*=3.04*x*+413.49	0.968	*y*=−0.11*x*+1663.83	0.894
*Corynoxeine*	*y*=2.36*x*+226.05	0.963	*y*=−0.10*x*+1180.52	0.908
The sum of peak areas	*y*=10.26*x*+1448.60	0.954	*y*=−0.30*x*+4109.15	0.907

## Data Availability

The data used to support the findings of this study are included within the supplementary information files.

## Supplementary Materials

Supplementary Materials The supplementary materials are the raw data of this experiment. From these data, Figures 4 and 5 can be obtained. Besides, Minitab was employed to analyse data to obtain Figure 6.

## References

[1] Lin B., Wang Y., Zhou Y., Zhao Z. (2013). Study on thin layer drying of *Rosa laevigata* michx. *Engineering*.

[2] Xu C.-J., Liang Y.-Z., Chau F.-T., Heyden Y. V. (2006). Pretreatments of chromatographic fingerprints for quality control of herbal medicines. *Journal of Chromatography A*.

[3] Liang X. M., Jin Y., Wang Y. P., Jin G. W., Fu Q., Xiao Y. S. (2009). Qualitative and quantitative analysis in quality control of traditional Chinese medicines. *Journal of Chromatography A*.

[4] Liu X., Wang X.-L., Wu L. (2014). Investigation on the spectrum-effect relationships of Da-Huang-Fu-Zi-Tang in rats by UHPLC-ESI-Q-TOF-MS method. *Journal of Ethnopharmacology*.

[5] Yang M., Wu J., Xu X., Jin Y., Guo Y., Chen J. (2006). A new lignan from the Jian-er syrup and its content determination by RP-HPLC. *Journal of Pharmaceutical and Biomedical Analysis*.

[6] Liang Y., Xie P., Chan K. (2004). Quality control of herbal medicines. *Journal of Chromatography B*.

[7] Liu M., Wu Y., Huang S., Liu H., Feng J. (2018). Spectrum–effect relationship between HPLC fingerprints and hypolipidemic effect of *Curcuma aromatic*. *Biomedical Chromatography*.

[8] Xu G., Xie M., Yang X. (2014). Spectrum-effect relationships as a systematic approach to traditional Chinese medicine research: current status and future perspectives. *Molecules*.

[9] Chinese Pharmacopoeia Committee (2015). *Chinese Pharmacopoeia*.

[10] Hao B., Yang X., Feng Y., Hong Y. (2014). Advances in studies on *Uncariae Ramulus Cum Uncis*Pharmaceuticals based on chemical stability. *Chinese Journal of Traditional Chinese Medicine*.

[11] Zhang L., Sun T., Cao Y. (2010). Hypotensive and diastolic blood vessel effect of *Uncariae Ramulus Cum Uncis*. *Pharmacology and Clinic of Chinese Medicine*.

[12] Huang R., Tan D., Zhang P., Huang B., Tao Y., Liu J. (2012). Analysis on *rhynchophylline* in *Uncariae Ramulus Cum uncis*from different habitats in Guangxi at various periods. *Chinese Traditional and Herbal Drugs*.

[13] Yang X., Feng Y., Wu F., Ruan K., Feng Y. (2013). Determination of *rhynchophylline* and *isorhynchophylline* in *Uncariae Ramulus Cum Uncis.* by HPLC. *Chinese Journal of Traditional Chinese Medicine*.

[14] Zhang R., Liu R., Liu Q., Nin S., Wang N. (2009). Extraction and determination of *rhynchophylline* and *isorhynchophylline* in *Uncariae Ramulus Cum Uncis*. *Traditional Chinese drug research and clinical pharmacology*.

[15] Jinag Y., Xu H. (2011). Determination the content of *rhynchophylline* and *isorhynchophylline* in *Uncariae Ramulus Cum Uncis* by ultra performance liquid Chromatography. *Chinese Journal of Traditional Chinese Medicine*.

[16] Chen C., Jin R., Li Y., Zhong J., Zhang H. (1999). Study on the antiplatelet aggregation and antithrombotic effects of *Rhynchophylline*. *Medical Research Communication*.

[17] Lu F., Lin B., Zou Lu., Lu X. (2017). Study on the constant temperature drying model of Uncaria and its kinetics. *Journal of Mountain Agriculture and Biology*.

[18] Chen Q. (1994). *Chinese Medicine Pharmacology Research Methodology*.

[19] Babalis S. J., Papanicolaou E., Kyriakis N., Belessiotis V. G. (2006). Evaluation of thin-layer drying models for describing drying kinetics of figs (*Ficus carica*). *Journal of Food Engineering*.

[20] Rafiee S., Sharifi M., Keyhani A. (2010). Modeling effective moisture diffusivity of orange slice. *International Journal of Food Properties*.

[21] Goyal R. K., Kingsly A. R. P., Manikantan M. R., Ilyas S. M. (2007). Mathematical modelling of thin layer drying kinetics of plum in a tunnel dryer. *Journal of Food Engineering*.

[22] Yang X., Feng Y., Wu F., Ruan K., Feng Y. (2013). Determination of *rhynchophylline* and *isorhynchophylline* in *Uncaria tomentosa* by HPLC. *Chinese Journal of Traditional Chinese Medicine*.

